# La Crosse virus spread within the mosquito population in Knox County, TN

**DOI:** 10.1371/journal.pone.0249811

**Published:** 2021-04-16

**Authors:** Cameron Cook, Annastashia Blesi, Samantha Brozak, Suzanne Lenhart, Hanna Reed, Cassandra Urquhart, Abelardo Moncayo, Rebecca Trout Fryxell

**Affiliations:** 1 Department of Mathematics, University of Tennessee, Knoxville, TN, United States of America; 2 Department of Higher Education and Student Affairs, Bowling Green State University, Bowling Green, OH, United States of America; 3 School of Mathematical and Statistical Sciences, Arizona State University, Tempe, Arizona, United States of America; 4 Department of Mathematics, University of Central Florida, Orlando, FL, United States of America; 5 Department of Entomology and Plant Pathology, University of Tennessee, Knoxville, TN, United States of America; 6 Vector-borne Disease Section Tennessee Department of Health, Nashville, TN, United States of America; Faculty of Science, Ain Shams University (ASU), EGYPT

## Abstract

In Appalachia, La Crosse virus (LACV) is a leading pediatric arbovirus and public health concern for children under 16 years. LACV is transmitted via the bite of an infected *Aedes* mosquito. Thus, it is imperative to understand the dynamics of the local vector population in order to assess risk and transmission. Using entomological data collected from Knox County, Tennessee in 2013, we formulate an environmentally-driven system of ordinary differential equations to model mosquito population dynamics over a single season. Further, we include infected compartments to represent LACV transmission within the mosquito population. Findings suggest that the model, with dependence on degree days and accumulated precipitation, can closely describe field data. This model confirms the need to include these environmental variables when planning control strategies.

## Introduction

La Crosse encephalitis (LACE) is the leading pediatric arboviral disease in the continental United States [1]. LACE typically affects children younger than 16 years, with the strongest prevalence in male children between the ages of 5-9 [1, 2]. Children diagnosed with LACE are infected with La Crosse virus (LACV), which generates symptoms such as headache, fever, behavioral changes, and seizures [3]. This wide variety of symptoms can cause LACE to go undiagnosed. When symptoms present severely enough for hospitalization, 12% of cases are discharged with neurological deficits that may cause behavioral changes over time [4]. Although originally found in the upper Midwest, LACE is primarily diagnosed in southern Appalachia [3, 5].

LACV is transmitted via the bite of an infected *Aedes* mosquito. In eastern Tennessee, *Aedes triseriatus* is the primary vector, while *Ae*. *albopictus* and *Ae*. *japonicus* are identified as accessory vectors [6–9]. LACV is maintained in nature through a zoonotic cycle between the vectors and amplifying-reservoir (e.g., scurrids) populations [10]; however, transovarial transmission (when a female mosquito oviposits infected eggs) [8], transstadial transmission (when an infected immature remains infected through adulthood), and venereal transmission (from male to female through mating) [11] also occur.

One of the first to work on LACV was DeFoliart [12], who focused on *Ae*. *triseriatus* as the primary vector in the upper Midwest, prior to the introduction of *Ae*. *albopictus*. Bewick et al. [13] used mathematical modeling (with a system of ordinary differential equations) to suggest that *Ae*. *albopictus* was not responsible for the LACE outbreak in southern Appalachia and that other environmental scenarios must be responsible. These studies helped lay the groundwork for others in the field. Vector-borne diseases are often heterogeneous across space and time, and this heterogeneity can be explained with data-driven modeling. It is known that data-driven models help develop surveillance programs for mosquito-borne diseases. (e.g., [14–16], West Nile virus ([17–20]), and even Zika virus ([21–24]).

Models strongly connected to La Crosse data are just beginning to be developed. Previously, Nance et al. [25] built a system of ordinary differential equations to model the fluctuations of the *Ae*. *albopictus* populations in a single East Tennessee season. This model used transition rates dependent on precipitation, temperature, and the rate of change of temperature. Their work involved data from the most abundant species, *Ae*. *albopictus*, and used some parameters described in Tran et al. [26] for the immature class (larvae and pupae). Ghatak et al. [27] then developed a model that incorporated temperature and accumulated precipitation to explain the dynamics of all three LACV vectors over a single season. The oviposition rate and transition rates from eggs to the immature class and then to the host-seeking class were estimated as functions of temperature and accumulated precipitation [27]. While both models were useful, these two models did not involve mosquitoes infected with LACV. Therefore, the goal of this project is to incorporate LACV infection with data-driven modeling to investigate *Aedes* mosquitoes and relate population dynamics to local environmental variables. We build on previous work by using entomological data from Urquhart et al. [28], with the temperature and precipitation data, as well as using temperature to find rates explicitly dependent on degree days for the stage transitions of the mosquitoes.

Using our mosquito and environmental data, we build a system of ordinary differential equations to represent a mosquito population with appropriate life-stage transitions connected to our data and biological mechanisms. After that, disease status features coupled with the life-stage mechanisms are incorporated into the model with the presence of LACV in the mosquitoes. After finding particular functional forms for the transition rates (depending on environmental data) and estimating appropriate model parameters by fitting to mosquito data, our corresponding simulation results can suggest possible management actions.

## Materials and methods

### Mosquito-data collection

This paper uses the collection data previously presented in Urquhart et al. [28]. Eight properties within Knox County, Tennessee were monitored weekly, of which five were previous LACV-positive case houses (doctor-diagnosed patient in 2011-2012), and three had no known previous incidence of LACV, but were within 5 km of the previously positive residencies (sites). This is currently the only dataset with weekly trap data with LACV results from Knox County.

Starting on 25 June and ending on 15 October 2013, trapping for adult mosquitoes included three host-seeking traps, a gravid trap, and a resting trap. Some results of trap collections by species and week are presented in Urquhart et al. [28]. When traps were retrieved, collecting equipment was removed and stored in coolers lined with ice packs to keep specimens alive for mosquito identification and to preserve viral RNA. In the laboratory, adults were aspirated from the collecting equipment, transferred to cups, and provided a 10% sugar-water source until exposed to triethylamine for identification. The triethylamine-exposed (now-paralyzed) mosquitoes were then identified to species and sex [29]. Identified mosquitoes were organized into pools of 25 or less specimens (one pool had 33 specimens), and separated by species, sex, and life stage. All samples were stored at -80 degrees Celsius to preserve viral RNA.

Two ovitraps also operated at each site because LACV is also transmitted transovarially [8]. Methods for ovitrapping were previously described in Urquhart et al. [28]. Ovitraps were black plastic cups filled with approximately 250 mL of distilled water with a 1 and 5/8 inch strip of egg paper (seed germination paper) attached to the inside of the cup. Weekly, egg papers and water were replaced. Only egg papers were collected, properly labeled (site, trap, date), then refrigerated for 1-2 weeks to allow the paper to dry and prevent any early hatchings; water with potential larvae were discarded. Mosquito eggs were counted and egg papers were stored at room temperature for 4-6 weeks until hatching. To hatch eggs, dried egg papers were submerged in 300 mL oak infusion water, given approximately 0.5 grams of ground dog food as a food source, and placed in an incubator at 31 degrees Celsius and 85 percent humidity on a 16 hour light cycle. Mosquitoes were observed daily and given additional food as needed. When adults emerged, they were treated as field-collected mosquitoes and stored at -80 degrees Celsius.

Pooled *Aedes* vectors of LACV were separated from other genera (i.e. *Anopheles, Psorophora, Mansonia*, etc.) and shipped overnight with ice packs to the Tennessee Department of Health Vector-borne Diseases laboratory (TNDOH). Mosquito pools were homogenized on a Retsch MM300 shaker for 90 sec, centrifuged at 5000 rpm for 5 minutes, and stored at -80 degrees Celsius. After purification with the QIAamp Viral Isolation 96 well protocol on the BioRobot 9604 or the QIAamp Viral RNA mini kit (Qiagen, Valencia, California), 5*μ*l of extract was used to screen for LACV using previously published protocols [30]. Minimum infection rates (MIR) of LACV were calculated by dividing the number of positive pools by the total specimens tested and multiplying by 1000.

Adult collections yielded 821 pools of *Aedes* LACV vectors of which eight (0.98 percent) of the *Aedes* pools were LACV positive and consisted of seven *Ae*. *triseriatus* (0.85%) and one *Ae*. *albopictus* (0.12%) pool. None of the 61 pools of *Ae*. *japonicus* tested positive. Additionally, none of the 306 pools of reared *Aedes* (from the immature population) tested positive for virus, but this may have been due to a freezer malfunction before these samples were screened. All of the LACV positive pools were females and were collected at five of the eight sites consisting of both previously LACV positive and unknown sites, from June through late September. Minimum infection rates ranged from 0 to 46.512 throughout the collection period. The calculated MIR for each vector was 17.59 for *Ae*. *triseriatus* (398 specimens) and 0.29 for *Ae*. *albopictus* (3486 specimens). Overall, the MIR for Knox county LACV vectors was 1.9985 (8 positives / 4003 LACv vectors x 1000); this was not significantly different from the 2012 *Ae*. *triseriatus* MIR of 1.331 in Union county (X2 = 0.285; df = 1; P = 0.5937) [30], but both were greater than the previous MIR of 0.26 in western North Carolina [2].

### Environmental data

Precipitation and temperature usually have effects on mosquito populations in life stage transitions and in oviposit rates [31, 32]. Mosquitoes lay their eggs in water, and those eggs hatch in water, making the accumulated precipitation a factor in the oviposition rate of the mosquito population. Also, for a mosquito to develop, it must spend a certain amount of time above a temperature threshold. The level and the time above this threshold are combined in the definition of degree days. [33–35] Due to these environmental factors, we collected weather data from sites that ranged across 7 zip codes in the Knox County area. In order to find the average site’s degree day data, we first import the hourly temperature information for the sites via the website Weather Underground [36]. Weather Underground [36] uses the temperatures from the closest airport weather station to a location for its historical weather records. The two airports we took data from were McGhee Tyson and Oak Ridge airport station. As two of the sites were in the zip code for the Oak Ridge airport station, and six were in the zip code for the McGhee Tyson airport station, we took an average of the data between the the two airports. The temperature information is manually imported per hour per day from 15 May 2013 to 19 October 2013. The temperature for the average site is then averaged between the Oak Ridge site and the McGhee Tyson site per hour. Using mosquito development results from [37–39] involving temperatures and several types of mosquitoes, we chose 15° C as the minimal development temperature for the threshold in degree days. The threshold is subtracted from the hourly temperature, and if that difference is positive, the difference contributes to the degree hours/day [40]. If the difference is negative, there is no contribution from that difference. For each hour of the day, we sum the differences for the hourly temperature above the temperature threshold,

$$\begin{array}{c} \sum_{i=1}^{24}\frac{{\left(Temperature_{i}-Threshold\right)}^{+}\left(1hour\right)}{24}. \end{array}$$

The precipitation data is gathered in a similar way. We use Weather Underground [36] to obtain the hourly precipitation data in centimeters for both of the referenced airport weather stations. The precipitation is averaged between the sites as described earlier and accumulated hourly to obtain the daily precipitation data that is used in the model.

### Mosquito population model

We divide our population at time *t* into four compartments: eggs *E*(*t*), immature *I*(*t*), host-seeking *H*(*t*), and gravid *G*(*t*), where each compartment describes the size of that population at time *t*. The immature compartment is composed of the larval and pupal stages; these two stages were lumped together due to lack of data to distinguish between them. Adult female mosquitoes enter a reproductive cycle of mating, blood-feeding, and ovipositing. We consider mosquitoes who are mating and blood-feeding as host-seeking *H*, while mosquitoes who are seeking to oviposit are gravid *G*. Thus, the *H* and *G* compartments only contain female mosquitoes. As temperature and precipitation have a significant effect on the mosquito population, we will choose functional forms for some of the transfer and transition rates in our model to accurately describe the dynamics of the mosquito population at an average trapping site in Knox County, Tennessee for the summer of 2013.

**Fig 1:**
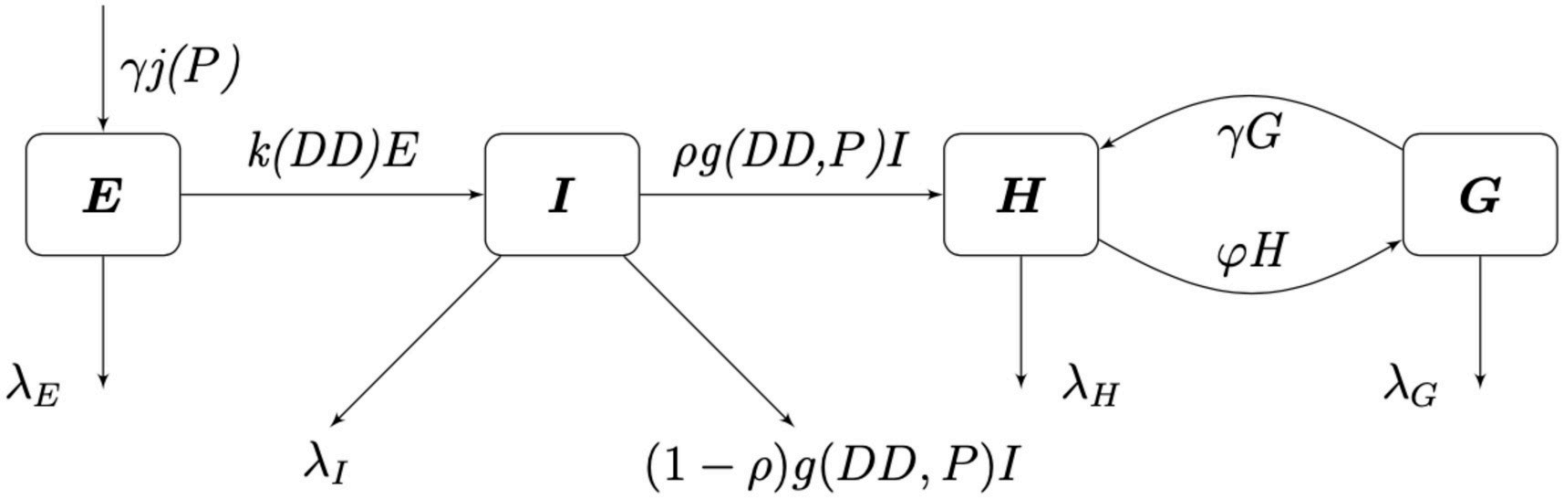
Flow diagram for mosquito population model.

Seen in our diagram below, Fig 1, we assume that *γ* is the rate at which gravid mosquitoes are ovipositing at time *t*, hence *γG* is the rate of ovipositing eggs. We assume that when a gravid mosquito oviposits, there are *j* eggs laid on average. Thus, *jγG* is on average the rate of the number of eggs laid by a gravid mosquito per day. With the development rate of eggs, *k*, and the immature development rate of immatures, *g*, we have that *kE* is the transition of eggs into immature and *gI* is the transition rate of immatures into adults. We assume that there is a constant sex ratio in the population, where *ρ* is the proportion of females in the population. Thus, *ρgI* is the transition rate of female immatures to host-seeking *H*, as we are only considering female mosquitoes in the host-seeking and gravid compartments. Note that (1 − *ρ*)*gI* is the rate of males leaving the *I* compartment, as the females transition to the *H* compartment. We have *φH* as the rate at which host-seeking mosquitoes finish mating/blood-feeding and become gravid *G*. Also, *γG* is the rate that gravid mosquitoes finish resting/ovipositing and become host-seeking *H*. We denote the death rates as λ_*E*_, λ_*l*_, λ_*H*_, and λ_*G*_ for eggs, immature, host-seeking, and gravid compartments, correspondingly.

We assume that the oviposition rate, *j*, of eggs depends on accumulated precipitation *P*, since mosquitos need standing water to lay eggs and to have eggs hatch. The development rate of eggs, *k*, depends on degree days *DD*, and the immature development rate, *g*, depends on both accumulated precipitation, *P*, and degree days, *DD*. The structure of the functional forms of *j*, *k* and *γ* will be chosen from the corresponding biological mechanisms and our environmental and mosquito data. Below is our system of differential equations for our population model associated with our diagram, Fig 1.

$$\begin{array}{c} \frac{dE}{dt}=j\left(P\right)\gamma G-(\lambda_{E}+k\left(DD\right))E \end{array}$$

$$\begin{array}{c} \frac{dI}{dt}=k\left(DD\right)E-(\lambda_{I}+g\left(DD,P\right))I \end{array}$$

$$\begin{array}{c} \frac{dH}{dt}=\rho g\left(DD,P\right)I-\left(\lambda_{H}+\varphi \right)H+\gamma G \end{array}$$

$$\begin{array}{c} \frac{dG}{dt}=\varphi H-(\lambda_{G}+\gamma )G \end{array}$$

### La Crosse transmission model

Our next task was to account for the presence of LACV in the mosquito population. We now add four compartments for the infected mosquitoes. We have 8 compartments: uninfected eggs *E*, uninfected immature *I*, uninfected host-seeking *H*, uninfected gravid *G*, infected eggs *E*_*v*_, infected immature *I*_*v*_, infected host-seeking *H*_*v*_, and infected gravid *G*_*v*_. We make the assumption that the mosquito populations longevity and behavior is unaffected by the presence of LACV. Hence, we assume that the rates for oviposition *j*, egg and immature development *k* and *g* respectively, host-seeking and gravid transition *φ* and *γ* respectively, and natural death λ_*E*_, λ_*I*_, λ_*H*_, and λ_*A*_ remain the same for both uninfected and infected populations.

**Fig 2:**
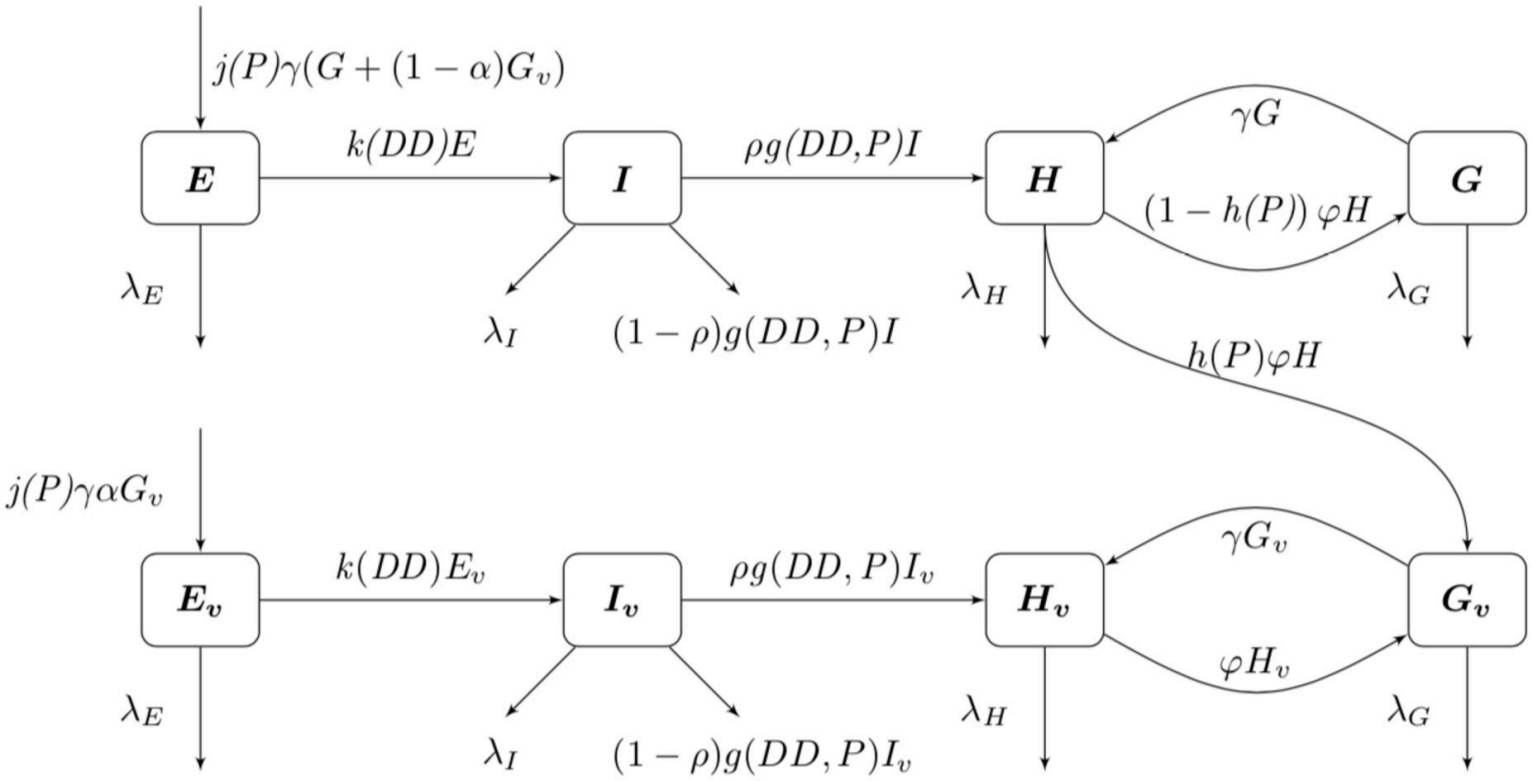
Flow diagram of La Crosse virus transmission model.

As LACV is transmitted through the mosquito population by means of maternal vertical transmission, we assume that when a gravid mosquito is infected with LACV and oviposits, a proportion of her eggs *α* is infected as well. Thus, at time *t*, the rates of oviposition are *αjγG*_*v*_ for infected eggs and *jγG*_*v*_ + (1 − *α*)*jγG*_*v*_ for uninfected eggs. LACV is maintained in the zoonotic cycle and venereal transmission is present as well [3]. In both of these transmission types, host-seeking mosquitoes have a chance of becoming infected through feeding on an infected host or venereal transmission, and thus we suppose that the rate of host-seeking mosquitoes becoming infected gravid is *hH*. The rate is denoted by *h*(*P*)*H* due to the effect of accumulated precipitation [26, 41]). The structure of *h*(*P*) and the value of *α* will be found through parameter estimation using our data. Our model with transmission is below, and the corresponding diagram, Fig 2.

$$\begin{array}{c} \frac{dE}{dt}=j\left(P\right)\gamma G+j\left(P\right)\gamma (1-\alpha )G_{v}-\left(\lambda_{E}+k\left(DD\right)\right)E \end{array}$$

$$\begin{array}{c} \frac{dI}{dt}=k\left(DD\right)E-(\lambda_{I}+g\left(DD,P\right))I \end{array}$$

$$\begin{array}{c} \frac{dH}{dt}=g\left(DD,P\right)\rho I-\left(\lambda_{H}+\varphi \right)H+\gamma G \end{array}$$

$$\begin{array}{c} \frac{dG}{dt}=(1-h(P))\varphi H-\left(\lambda_{G}+\gamma \right)G \end{array}$$

$$\begin{array}{c} \frac{dE_{v}}{dt}=j\left(P\right)\alpha \gamma G_{v}-\left(\lambda_{E}+k\left(DD\right)\right)E_{v} \end{array}$$

$$\begin{array}{c} \frac{dI_{v}}{dt}=k\left(DD\right)E_{v}-\left(\lambda_{I}+g\right)I_{v} \end{array}$$

$$\begin{array}{c} \frac{dH_{v}}{dt}=g\left(DD,P\right)\rho I_{v}-\left(\lambda_{H}+\varphi \right)H_{v}+\gamma G_{v} \end{array}$$

$$\begin{array}{c} \frac{dG_{v}}{dt}=h\left(P\right)\varphi H+\varphi H_{v}-\left(\lambda_{G}+\gamma \right)G_{v} \end{array}$$

## Results/discussion

### Population model results and discussion

As seen in Table 1, using data fitting, we estimated all of the parameters in the population model, except for *ρ* = 0.5, the assumed ratio of females in the population. To represent the impact of accumulated precipitation and degree days on the mosquitoes in our population model, we needed to find functional forms for *j*, *k* and *g*. To find those functional forms, we tested many combinations of functional responses, using polynomials, logistic functions, and convex combinations of functions. For each set of functional forms, we found the best fit parameters. We estimated the parameters using fmincon for constrained optimization in conjunction with MultiStart, which searches for multiple local minima. Our objective function was set as the relative error. Our error function to be minimized is the relative sum of squared residuals (difference between data and corresponding simulated values) of the MATLAB simulations from the field data which includes egg and adult data. The initial goal was to have the sum of the host-seeking and gravid compartments fit to the total adult data, but due to the sparseness of gravid mosquito collection, we only fit the host-seeking simulations to the host-seeking data. Egg simulations are fit to the egg data provided by the Trout Fryxell lab. Our objective function was set as the relative error, and we then choose the functional forms with the best fit over all our choices.

**Table 1:** Best fit parameters for mosquito population model.

Parameter	Value	Unit	Biological Meaning
*ρ*	0.5	unitless	ratio of females in population
λ_*E*_	0.10	day^−1^	egg death rate
λ_*I*_	0.12	day^−1^	immature death rate
λ_*A*_	0.07	day^−1^	adult death rate
*φ*	0.50	day^−1^	rate of *H* to *G*
*γ*	0.50	day^−1^	rate of *G* to *H*
*β*	65.16	eggs	maximum oviposition rate
*d*_*E*_	0.23	day^−1^	maximum devevelopment rate
*d*_*i*_	0.14	day^−1^	maximum development rate
*a*	134.57	centimeters	half saturation constant
*b*	65.36	(degree days)^2^	half saturation constant
*c*	0.89	unitless	weighting constant
*d*	127.06	(degree days)^2^	half saturation constant
*e*	168.21	centimeters^2^	half saturation constant

After many trials a variety of functional forms, we determined specific functional forms and coefficient values for oviposition, egg development, and immature development that yielded the best results. We utilized a Michaelis-Menten equation to describe the oviposition rate, a Holling III to describe the egg development, and a convex combination of a degree-day dependent Holling III function and an accumulated precipitation dependent Michaelis-Menten equation to describe the immature development rate. Since accumulated precipitation was significant in previous papers, it is logical to assume that it will take a little time for the rain to submerge the eggs and for them to hatch. Comparing the relative errors using accumulated precipitation over 3, 5, 7, 12, 14, 20, 25, 28 days, we found that using precipitation accumulated over 20 days resulted in the best approximation to the observed days. Below is the oviposition rate, *j*, of eggs, which is precipitation dependent; the development rate of eggs, *k*, which is degree day dependent; and the immature development rate, *g*, dependent on both accumulated precipitation, *P*_20_ over 20 days, and degree days, *DD*.

$$\begin{array}{c} j(P_{20})=\frac{\beta P_{20}}{a+P_{20}}\;k\left(DD\right)=d_{E}[\frac{DD^{2}}{b+DD^{2}}] \end{array}$$

$$\begin{array}{c} g(DD,P{}_{20})=d_{I}[c(\frac{DD^{2}}{d+DD^{2}})+\left(1-c\right)(\frac{P_{20}}{e+P_{20}})] \end{array}$$

Due to under sampling in the first week of the field data by the Trout-Fryxell lab [28], our simulations begin in week 2 of collection. We take our initial conditions from the data, starting with the 176^th^ day of the year:

$$\begin{array}{c} E(176)=228,\;H(176)=13. \end{array}$$

We assume that there were 110 immatures, roughly half of the egg population, and as there were more host-seeking mosquitoes in our data than gravid, we assume there were 7 gravid mosquitoes, giving

$$\begin{array}{c} I(176)=110,\;G(176)=7. \end{array}$$

**Fig 3:**
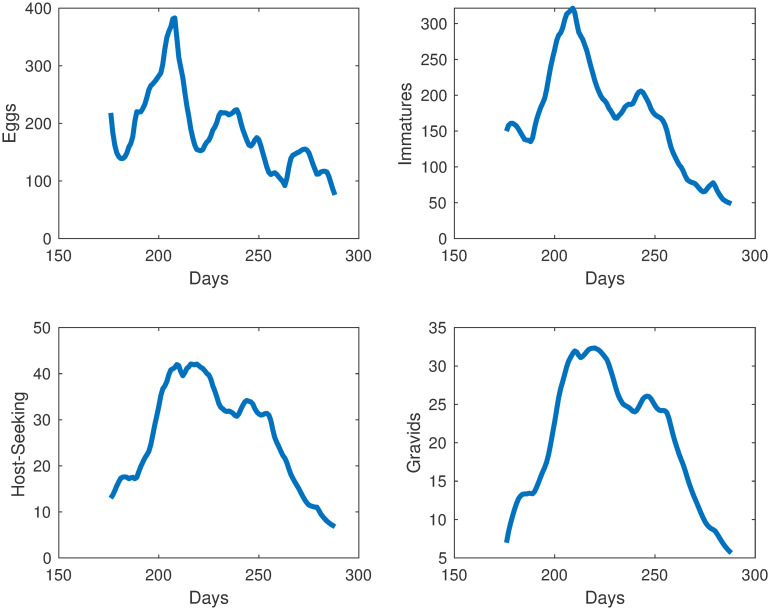
Simulations of mosquito population at different life stages in Knoxville, TN.

**Fig 4:**
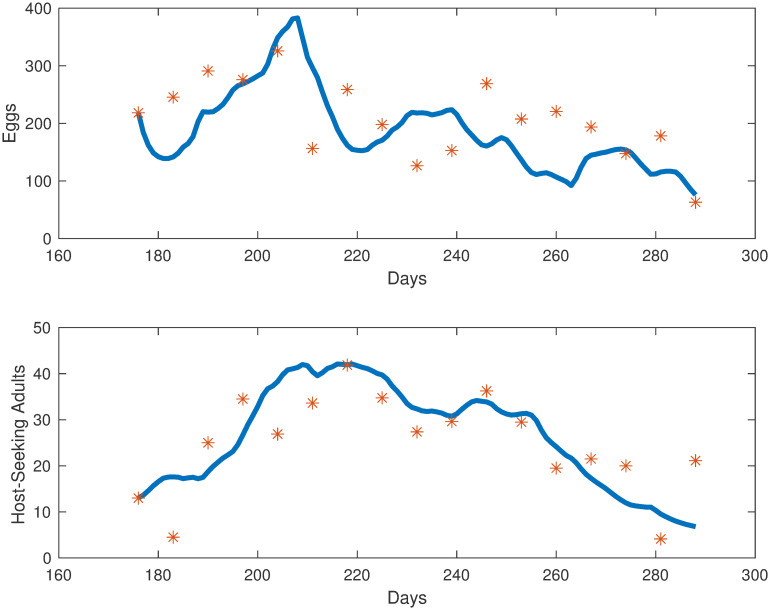
Collected egg and adult mosquito data (represented by red stars) versus egg and adult model simulations (represented by the blue line).

The simulations for each compartment are shown in Fig 3. We plot the total number of individuals in each compartment versus time, which started in late June, going through early October. Also in Fig 4, we show the egg and adult data versus the output from our model. The error we obtained for our model was 11.94%.

### Infection model results and discussion

For our model describing infection, we used the estimated parameters from our mosquito population model, with one new rate and one new functional form left to determine. We estimated the parameters to minimize the relative error using fmincon for constrained optimization in conjunction with Multistart. We hypothesize that the proportion of host-seeking mosquitoes becoming infected gravid is affected by the accumulated precipitation. After testing multiple forms and considering how this interaction is affected by accumulated precipitation, we decided on a linear functional form dependent on accumulated precipitation. After observing the data, we noticed that spikes in infection corresponded to higher levels of accumulated precipitation over a duration of 3 days compared to other days of accumulated precipitation. Thus, we define a functional form, *h*(*P*_3_) = *fP*_3_, to describe the transmission of LACV to host-seeking mosquitoes dependent on 3 days of accumulated precipitation. Note that here we use 3 days of accumulation precipitation as our dependence whereas for the development of the mosquitoes we use 20 days of accumulation. Using the data with Multistart and fmincon, we estimated *α*, the ratio of infected eggs oviposited. See Table 2 for the values of the 2 new parameters for this model.

**Table 2:** Parameters used in La Crosse infection model simulations.

Parameter	Value	Unit	Biological Meaning
*α*	0.01	unitless	ratio of infected eggs laid
*f*	0.06	unitless	infection constant

**Fig 5:**
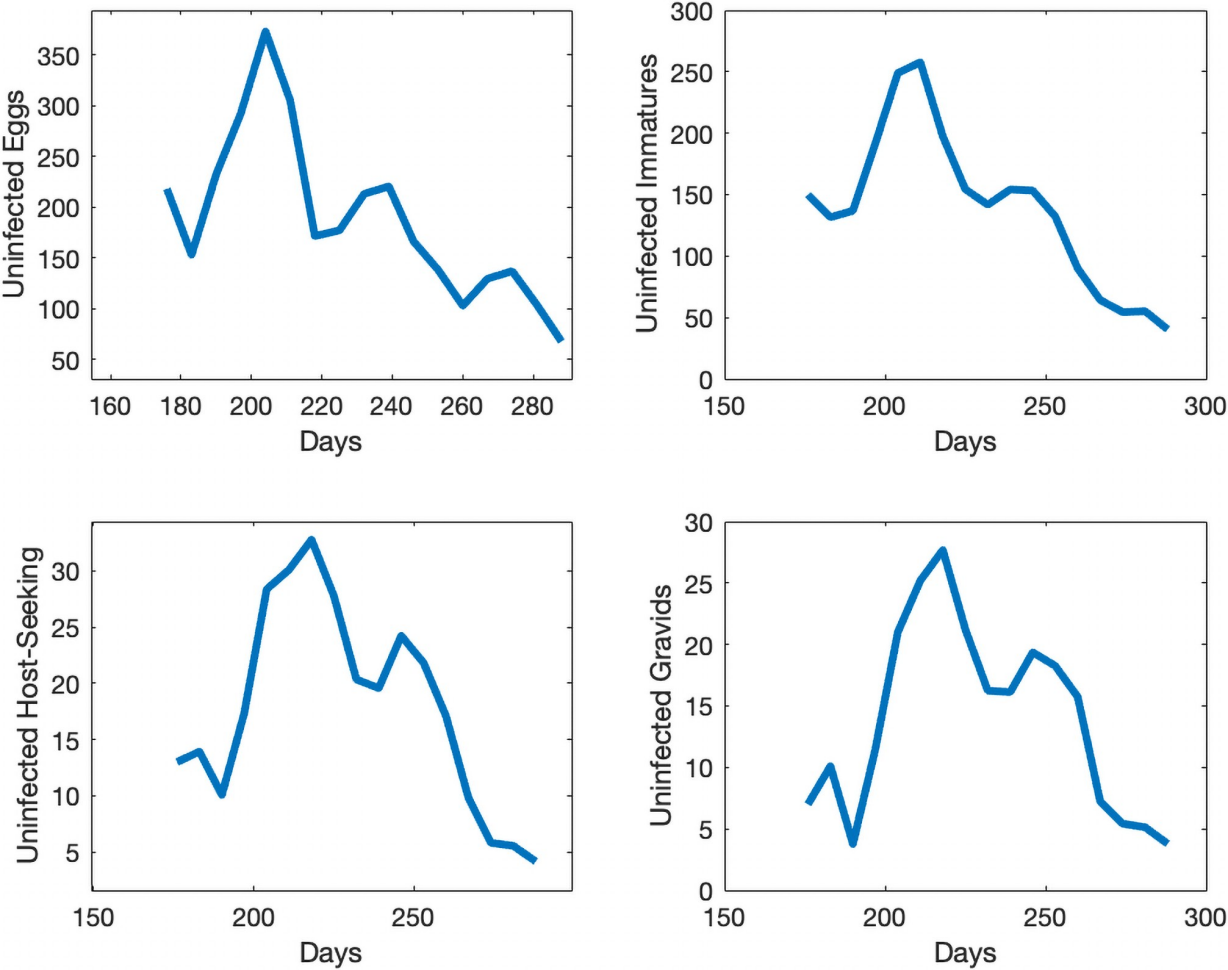
Simulations of individuals in the uninfected compartments for the infection model at different life stages in Knoxville, TN.

**Fig 6:**
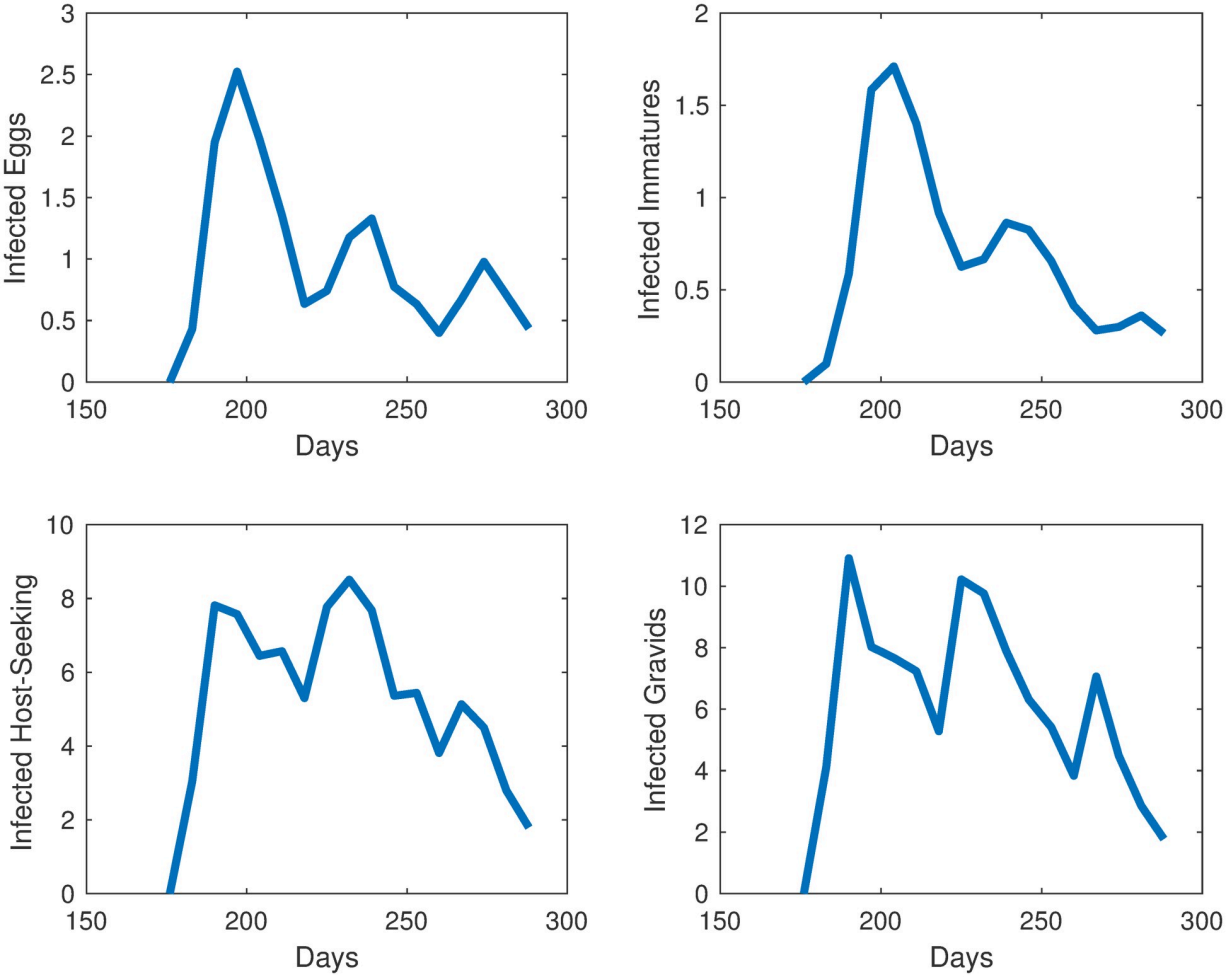
Simulations of individuals in the compartments for the infection model at different life stages in Knoxville, TN.

**Fig 7:**
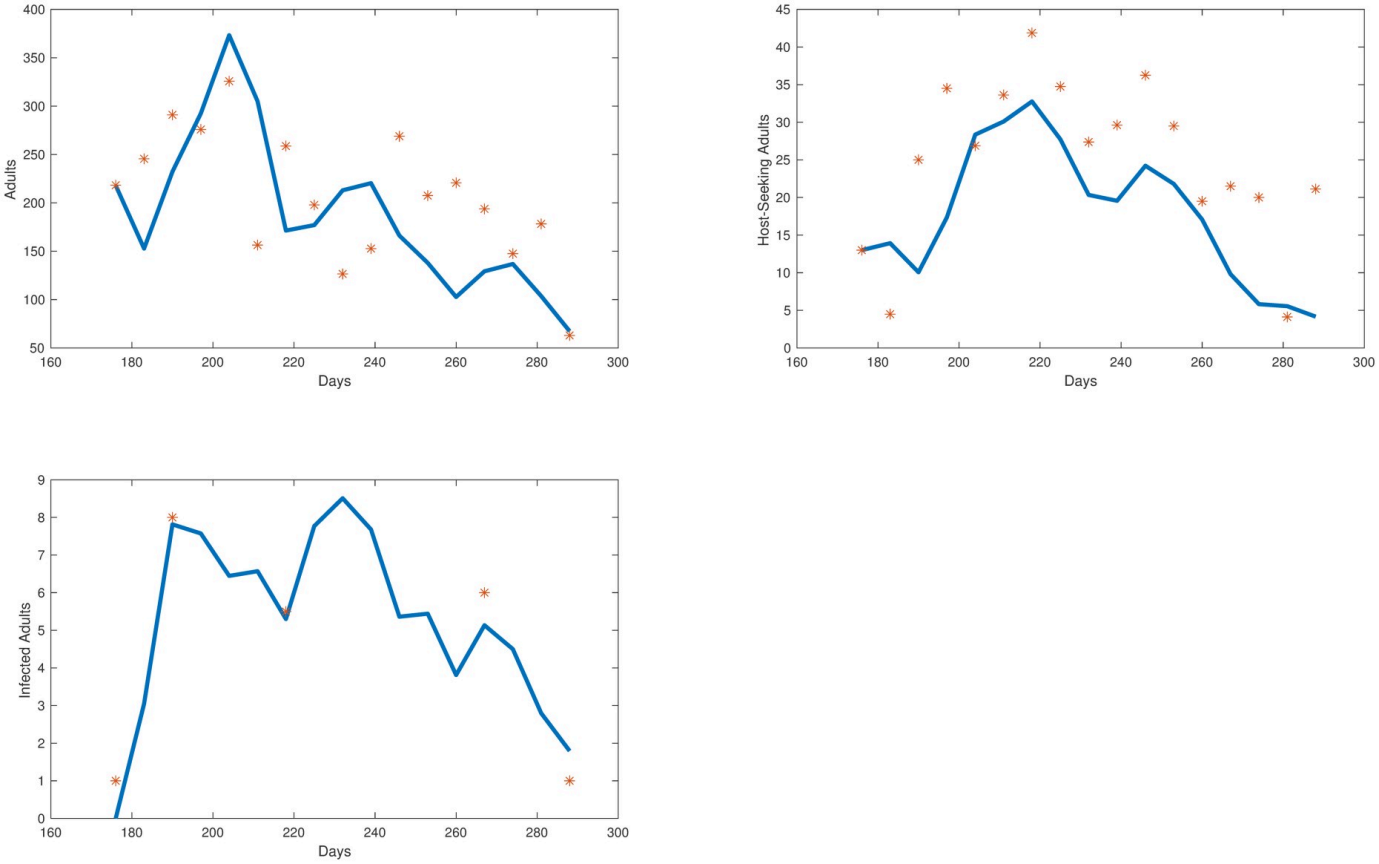
Collected egg, uninfected adult, and infected adult mosquito data (represented by red stars) vs egg, uninfected adult, and infected adult model simulations (represented by the blue line).

We take the same initial conditions from the mosquito population model and assume there are no individuals in any of the 4 infected compartments to start, as infection is not present in the first collection week data. Our objective function is set equal to our relative error. Our error to be minimized is the relative sum of squared residuals of the MATLAB simulations from the field data which includes egg, uninfected adult, and infected adult data. Due to the costs associated with testing individual mosquitoes and the likelihood of detecting LACV, the mosquito populations were pooled, and we assume that if a pool had LACV present then half of the mosquitoes in that sample were infected. We cannot assume all mosquitoes were LACV positive; we chose 50% as the average between 1 and 100%. Over the course of the study, few LACV-positive pools were noted, but their lack of detection does not indicate their absence from the population. The simulations for each compartment are shown in Figs 5 and 6 We plot the total number of individuals in each compartment versus time, which started in late June, going through early October. Also, in Fig 7 we show the collected egg, uninfected host-seeking adult, and infected adult data versus the output from our model. The error we obtained for our model was 27%.

## Conclusion

Building on two previous models using data in Knox County [25, 27], we developed a non-autonomous system of ordinary differential equation to represent mosquito populations with the transmission of LACV in Knox County, Tennessee. Our model goes beyond those two mosquito population models to include the infected mosquito compartments and disease progression dynamics. The format of the dependence on temperature and precipitation and the rates in our model were driven by biological mechanisms and the data from Urquhart et al. [28]

We started our model with the mosquito population at an average trapping site. By fitting our model to field data collected by the Trout Fryxell laboratory, we were able to estimate parameters with MATLAB and simulate a mosquito population with 11.94% error. Investigating many functional forms, we determined that a precipitation dependent oviposition rate, a degree-day dependent egg development rate, and both a precipitation and degree day dependent immature development rate led to the best fit. We found for the population that we were considering, accumulated precipitation over a duration of 20 days yielded the least relative error.

After building our population model, we devised a mosquito population model that incorporated the transmission of LACV among the mosquito population. Using infective pool data collected by the Trout Fryxell laboratory, we were able to estimate two more parameters to simulate a mosquito population with LACV present with 27% error. Our simulations imply that LACV is maintained in the mosquito population and that there are occasional spikes in infection. The occasional spikes of infection are likely explained with the heterogeneity of the environment such as environmental conditions (rainfall, temperature), abundance of oviposition sites, host availability, and prevalence of infected hosts at a site. Finding better ways to represent the infected pools in models is an important next step.

The results of our model can be used as a resource for pest management and public health officials in decision making regarding several factors. The impact of accumulated precipitation on both models is a signal that prevention of standing water and drainage improvement is of the utmost importance in areas with a higher LACV threat. Our models show that the accumulated precipitation not only plays a significant role in the mosquito life cycle, but also plays a role in the transmission of LACV from infected mosquitoes. These models can also benefit health officials and pest management professionals in determining what time of year and what circumstance lead to increased mosquito population as well as when LACV is most prevalent in the mosquito population. While mosquito prevention is typically done throughout the summer, and health officials always encourage protection against mosquitoes, these models provide information as to when alert the general population to high threats.

We assume that the virus has no effect on the longevity or fecundity of mosquitoes. In our model, we have simplified the process by which LACV spreads throughout the mosquito population. We make the assumption that transovarial transmission can be described accurately by a constant infected proportion of eggs laid by infective gravid. We also use an accumulated precipitation dependent term, *h*, to describe the proportion of host-seeking mosquitoes which will have contracted LACV by the time they become gravid.

We used environmental variables with available data in our model structure. Future models could incorporate more local site-specific demographics/variables such as number of containers (with water, with larvae, with pupae, etc.). There is a lack of data available on transvenereal transmission (dependent on the infected male population) and zoonotic transmission (dependent on infected scurrid population). Future research should be conducted to understand these additional transmission mechanisms.

Over the duration of the collection period, a large proportion of the infective pools occurred at the beginning of collection. If collection began earlier, we could do more investigation into factors that contribute to the amplification of LACV in the scurrid population by observing the initial spikes of infection. In the future, more information about prevalence of LACV in the mosquito population may be needed in order to represent the corresponding percentage of positive mosquitoes in a positive pool.

## Supporting information

S1 Data (PDF)
