# Supplementary material for: La Crosse virus spread within the mosquito population in Knox County, TN
Source: PLoS One. 2021 Apr 16;16(4):e0249811. doi: 10.1371/journal.pone.0249811 (PMC8051795; doi:10.1371/journal.pone.0249811)
Supplement: S1 Data — (PDF) [file pone.0249811.s001.pdf]

This data is connected to C. Cook et al. in, La Crosse Virus Spread in Mosquito Population in Knox County, TN.

Week<br><br>(Week 1 corresponds to June 25th)	Adult Mosquito Averages (over 8 trapping sites)	Egg Mosquito Averages (over 8 trapping sites)	Infected Adult Mosquito Estimates (Half the number of mosquitos in the infected pools)
1	13	218.25	1
2	4.5	245.5	
3	25	291	8
4	34.5	275.875	
5	26.875	325.75	
6	33.625	156.375	
7	41.875	258.75	5.5
8	34.75	197.875	
9	27.375	126.5	
10	29.625	152.715	
11	36.25	268.875	
12	29.5	207.5	
13	19.5	220.75	
14	21.5	193.75	6
15	20	147/5	
16	4.125	178.25	
17	21.125	63	1

*Environmental Data was obtained from https://wunderground.com
